# Letting go of the physical exam: embracing telehealth solutions to oncology

**DOI:** 10.20517/2394-4722.2020.06

**Published:** 2020-04-28

**Authors:** Michael J. Overman

**Affiliations:** Department of Gastrointestinal Medical Oncology, The University of Texas M. D. Anderson Cancer Center, Houston, TX 77030, USA.

Despite the diminishing value of the physical exam in the management of many chronic diseases, it still represents a core component of clinical office visits^[1]^. In part, this reflects the reimbursement landscape that continues to support its use at every clinical encounter. However, as healthcare pivots towards a new focus on value-based care, it is imperative that we move beyond the confines of the in-person clinical encounter and embrace the tremendous potential for telehealth solutions to healthcare delivery.

The management of chronic diseases frequently relies on the subjective component of care represented by symptom management or the provision of information. In addition, in many chronic diseases, new diagnoses are made based on objective measures of laboratory or imaging testing. This is particularly true in medical oncology, where symptoms generally reflect well-defined toxicity profiles from anti-cancer systemic therapy or the space-occupying effects of metastatic disease, which in almost all settings would be diagnosed via imaging testing. In a medical oncology clinic, the two most common visit types are by patients undergoing active systemic anti-cancer therapy or surveillance for potential cancer recurrence.

In the first setting, medical oncology providers spend their time managing various symptoms such as nausea, fatigue, depression, anxiety, diarrhea, or pain. In addition, at almost all of these visits, laboratory studies are conducted to provide adequacy of blood counts for treatment and chemistries to assess dehydration. The critical aspects of these visits are subjective symptom assessment, laboratory review, education related to symptom management, and the prescription of symptomatic medications and anti-cancer therapy. The physical exam however, frequently represents time that is not allocated for symptom management education. Without the need to see each patient in person, patients could have laboratory testing, vital signs testing, and telehealth visits better integrated into their schedules. In fact, telehealth experiences in other internal medicine subspecialties have demonstrated a reduction in driving time and missed work^[2]^. As well, systemic anti-cancer therapy represents a recurring event in patients’ lives so optimization of their time should be our focus.

In the second context of surveillance, the physical exam is not needed when recent diagnostic imaging such as computed tomographic scans have just been conducted. In addition, when tumor markers are normal and a patient does not have new symptoms, a physical exam is of questionable value.

In essence, by tailoring in person clinic visits towards when there is highest need, we can help to integrate our delivery of care into the lives of our patients better. In medical oncology where patients’ lives are often measured in months or years, this improved integration would reflect a higher quality of life away from the medical system for our patients. This change in approach would also align with where many medical oncologists feel we need to be spending our time: educating our patients about symptom management and their cancer journey. Such time spent on cancer prognosis, end-of-life experience, clinical-trial understanding, symptom prevention and management should reduce the downstream utilization of high cost emergent care facilities and hospitalizations during and at the end of life. The ability to tailor visits to the needs of patients would enable in person visits to be reserved and dedicated to the critical discussions of end-of-life care planning, prognostication discussions or restaging discussions.

Making such changes to a medical oncology clinic to be fully integrated with telehealth delivery will not be easy as technological, liability, financial, and logistic scheduling challenges will exist^[3]^. In addition, the physical examination still has a symbolic meaning to patients as an acknowledgment of their complaints and an expected part of a physician office visit^[4]^. Studies have also demonstrated that the physical examination is a way for physicians to build empathetic relationships with their patients^[5]^. These concerns will need to be addressed and discussed with patients as telehealth moves forward. Could open acknowledgment of the missing aspect of touch and examination be a mechanism to attenuate this loss?

As we move towards a vision where we optimally integrate our practice with the lives of our patients, we must first agree to move away from our historical reliance on the physical examination. It is time taken not only to optimize a patients’ time at the end of life, but also to optimize their time throughout the entire cancer care journey.
